# Supplementary material for: In Vitro and Preclinical Antitumor Evaluation of Doxorubicin Liposomes Coated with a Cholesterol-Based Trimeric β-D-Glucopyranosyltriazole
Source: Pharmaceutics. 2023 Dec 11;15(12):2751. doi: 10.3390/pharmaceutics15122751 (PMC10747952; doi:10.3390/pharmaceutics15122751)

Synthesis of the cholesterol-based trimeric β-D-glucopyranosyltriazole

The compound was prepared according to Silva et al, 2018, as shown in the scheme below.

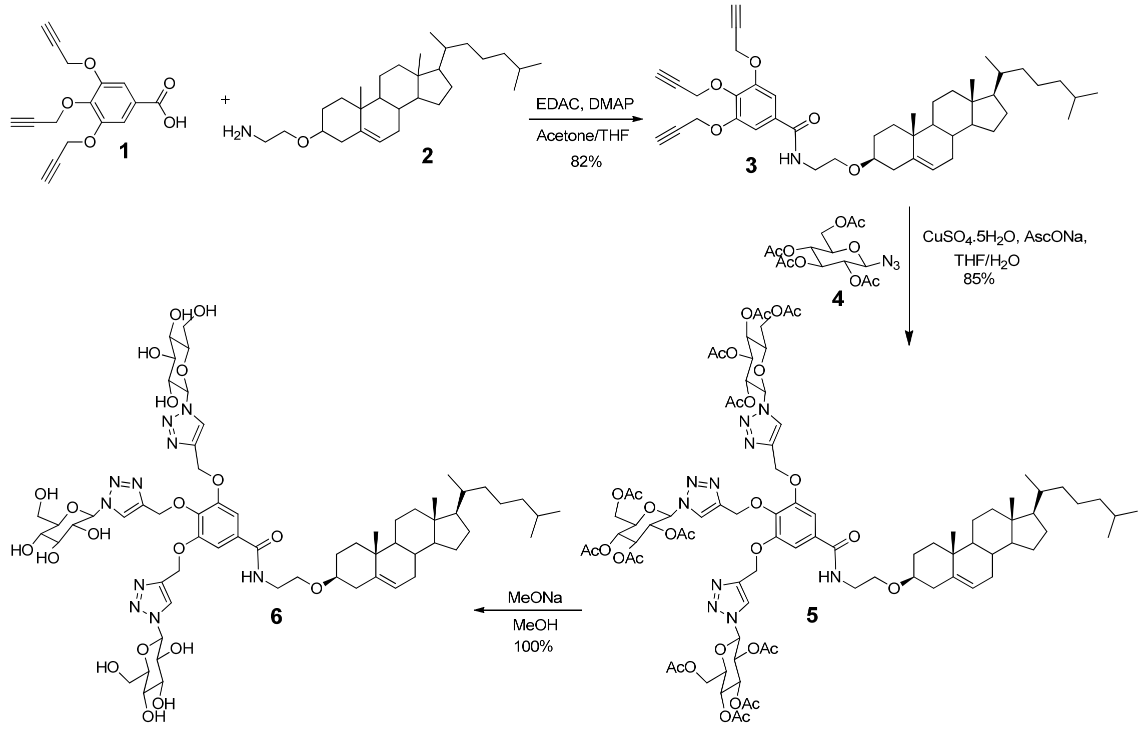

3,4,5-tris(propargyloxy)benzoic acid (**3**) (150 mg, 0.21 mmol), peracetylated β-D-glucopyranosyl azide (**4**) (279 mg, 0.75 mmol), $CuSO_4$ 10% w/v (0.5 mL 0.19 mmol) and sodium L-ascorbate (38 mg, 0.19 mmol) were stirred at room temperature for 4h in THF/water. After confirming the completion of the reaction on TLC (Hex: EtOAc = 1:1) the reaction mixture was concentrated in vacuo to give a crude product, which was purified by column chromatography over silica gel using EtOAc:Hex as the eluent to afford peracetylated trimeric β-D-glucopyranosyltriazole (**5**) a white powder. Yield: 333 mg, 85% (white powder). ^{1}H NMR (400 MHz, $CDCl_3$, δ): 8.49 (s, 2H), 8.39 (s, 1H), 7.09 (s, 2H), 6.91 (s, 1H), 6.24 (d, J = 9.5 Hz, 1H), 5.97 (d, J = 8.9 Hz, 2H), 5.64 (t, J = 8.9 Hz, 1H), 5.28-5.52 (m, 15H), 4.06-4.36 (m, 9H), 3.51-3.61 (m, 4H), 3.17-3.22 (m, 1H), 0.85-2.39 (m, 76H), 0.68 (s, 3H). ^{13}C NMR (100 MHz, $CDCl_3$, δ): 170.7, 170.1, 169.7, 166.2, 151.9, 144.9, 144.8, 142.0, 130.8, 123.1, 122.4, 122.2, 106.4, 86.0, 85.6, 79.6,

75.2, 75.0, 73.2, 72.9, 70.9, 70.3, 68.0, 66.8, 65.8, 62.93, 62.91, 61.8, 56.9, 56.3, 50.3, 42.4, 40.6, 39.9, 39.6, 39.2, 37.3, 37.0, 36.3, 35.9, 32.0, 29.8, 28.6, 28.3, 28.1, 24.4, 23.9, 22.9, 22.7, 21.2, 20.8, 20.70, 20.65, 20.3, 20.1, 19.6, 18.9, 12.0. Mass spectrum (MALDI-TOF) calculated for $C_{87}H_{118}N_{10}O_{32}Na$: 1837,781; found: 1837,477.

Peracetylated trimeric β-D-glucopyranosyltriazole (**5**) (204 mg, 0.11 mmol) was added to 10 mL of sodium methoxide at 0 °C. The resulting mixture was stirred at 0 °C for 1h and monitored by TLC (EtOAc). After confirming the completion of the reaction the reaction mixture was neutralized by an ion-exchange resin (Amberlite IRA 120 H^+), filtered, and evaporated, obtaining the deprotected final products which were used in the preparation of the liposomes. Yield: 147 mg, 100% (white crystals). ^{1}H NMR (400 MHz, DMSO-d_6, δ): 8.50 (s, 1H), 8.45 (s, 2H), 8.27 (s, 1H), 7.42 (s, 2H), 5.59 (d, J = 9.2 Hz, 2H), 5.54 (d, J = 9.2 Hz, 1H), 3.28-5.42 (series of m, 41H), 3.15 (m, 1H), 0.83-2.50 (m, 40H), 0.65 (s, 3H). ^{13}C NMR (100 MHz, DMSO-d_6, δ): 165.6, 151.7, 143.1, 142.6, 140.5, 139.1, 129.9, 123.9, 123.8, 121.2, 106.9, 87.6, 87.5, 80.0, 79.9, 78.5, 77.0, 72.09, 71.98, 69.6, 69.5, 65.9, 65.4, 62.3, 60.83, 60.76, 56.3, 55.7, 49.7, 41.9, 39.8, 39.2, 39.0, 38.8, 36.7, 36.3, 35.7, 35.2, 31.44, 31.38, 28.1, 27.8, 27.4, 23.9, 23.3, 22.6, 22.4, 20.6, 19.1, 18.6, 11.7. Mass spectrum (MALDI-TOF) calculated for $C_{63}H_{94}N_{10}O_{20}Na$: 1333,654; found: 1333,772

Infrared spectrum of compound 5

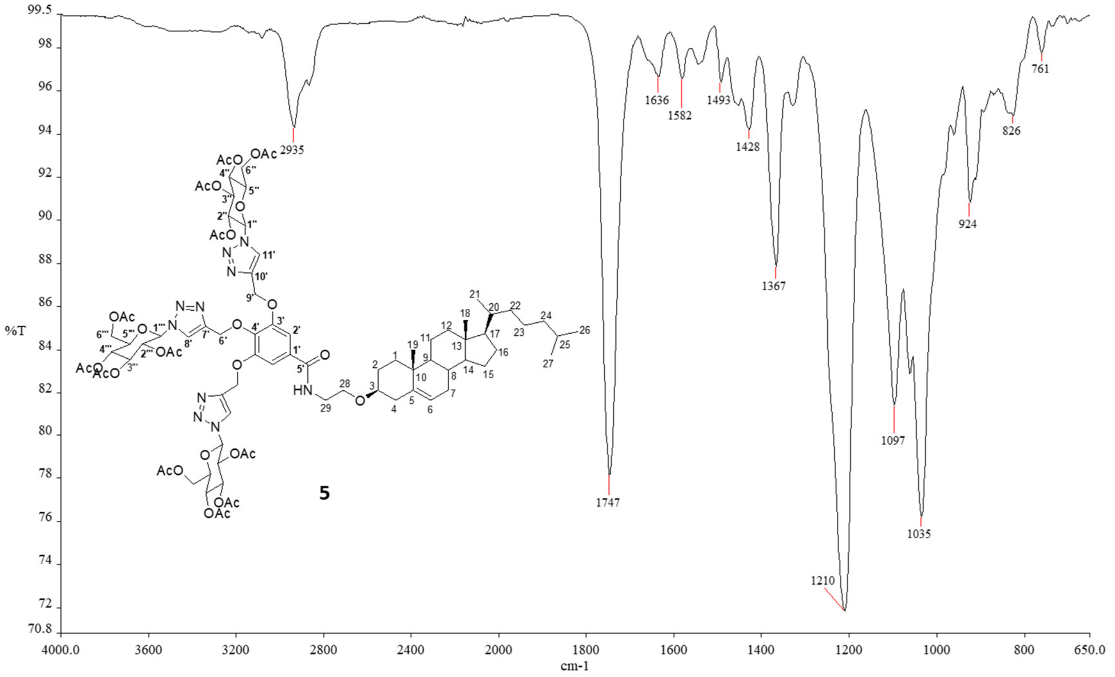

^{1}H NMR spectrum of compound 5 (400 MHz, $CDCl_3$)

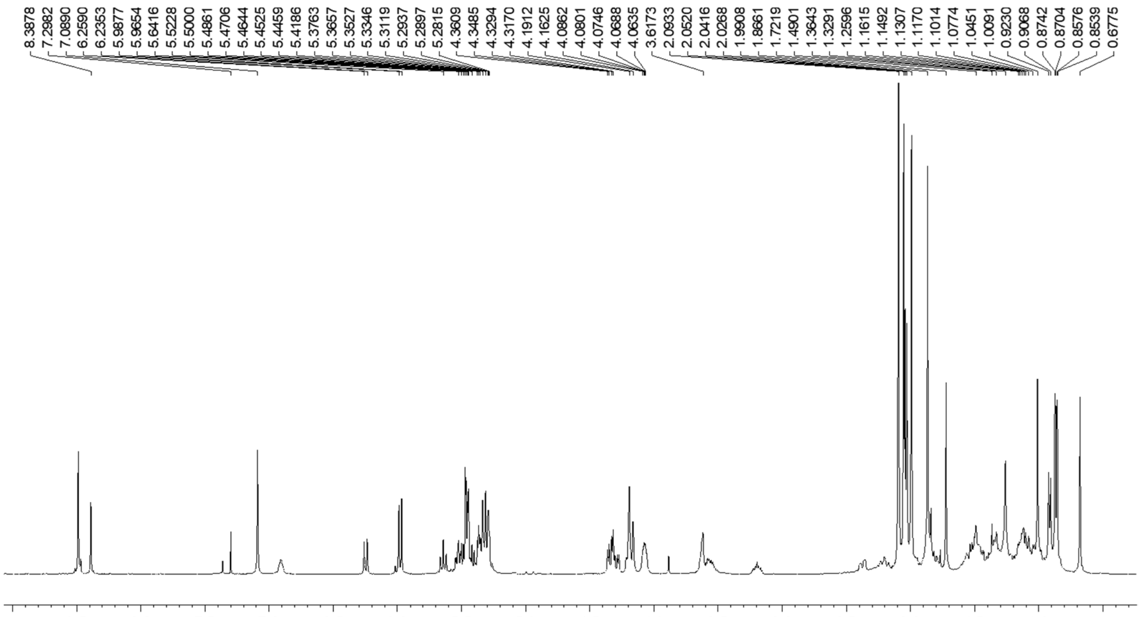

^{13}C NMR spectrum (top) and DEPT-135 (bottom) of compound 5 (100 MHz, $CDCl_3$)

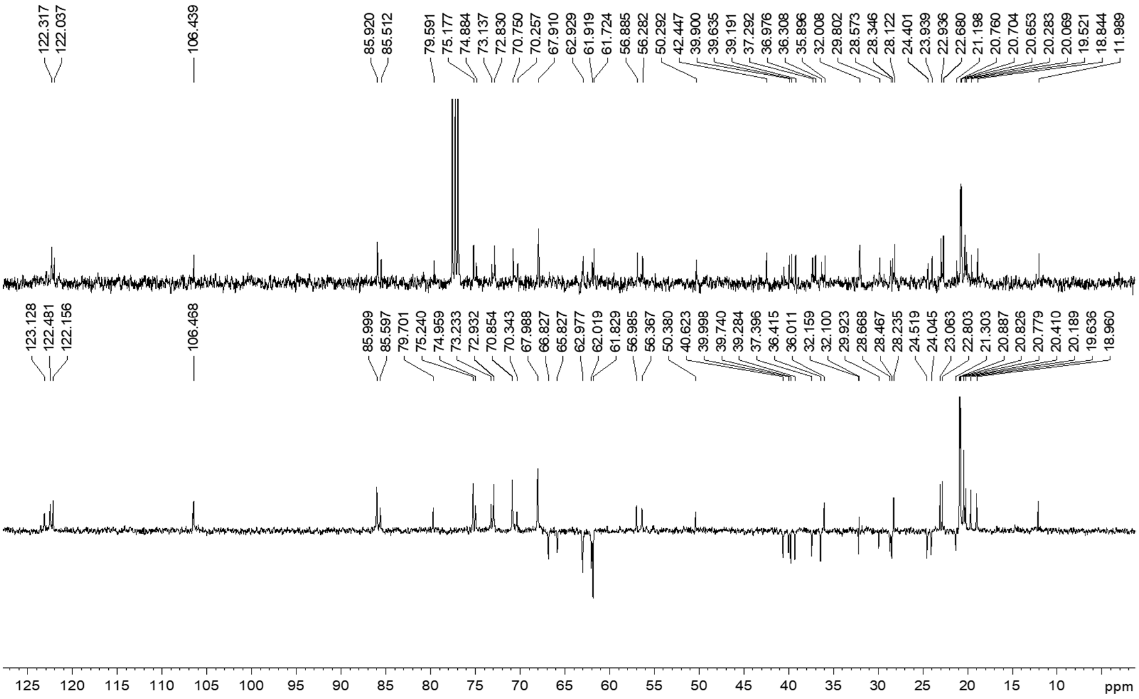

Mass spectrum (MALDI-TOF) of compound 5

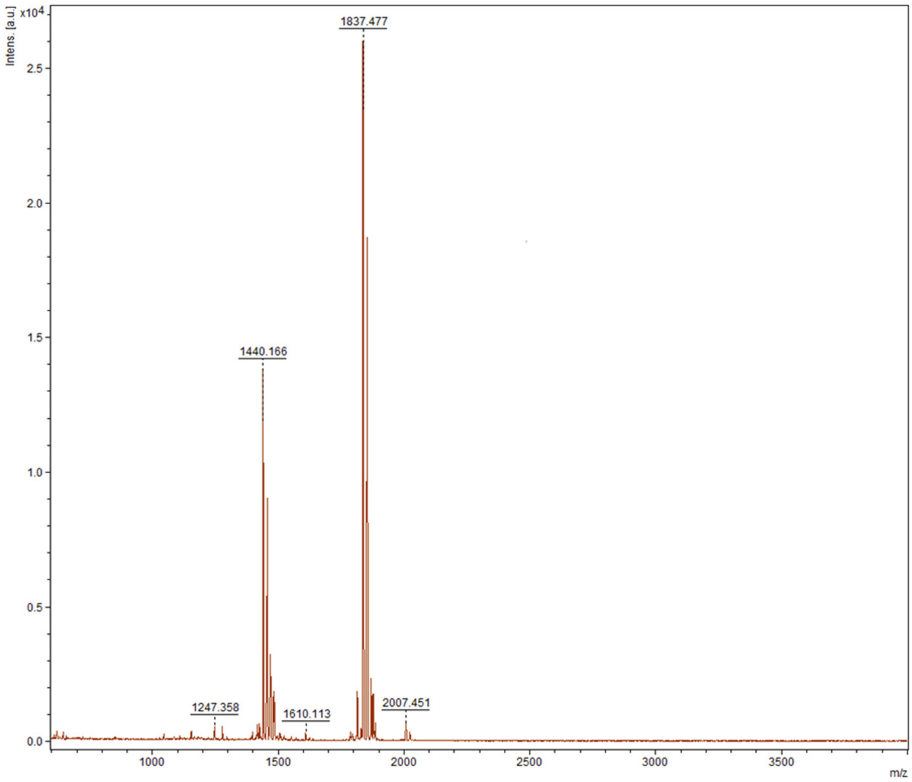

Infrared spectrum of compound 6

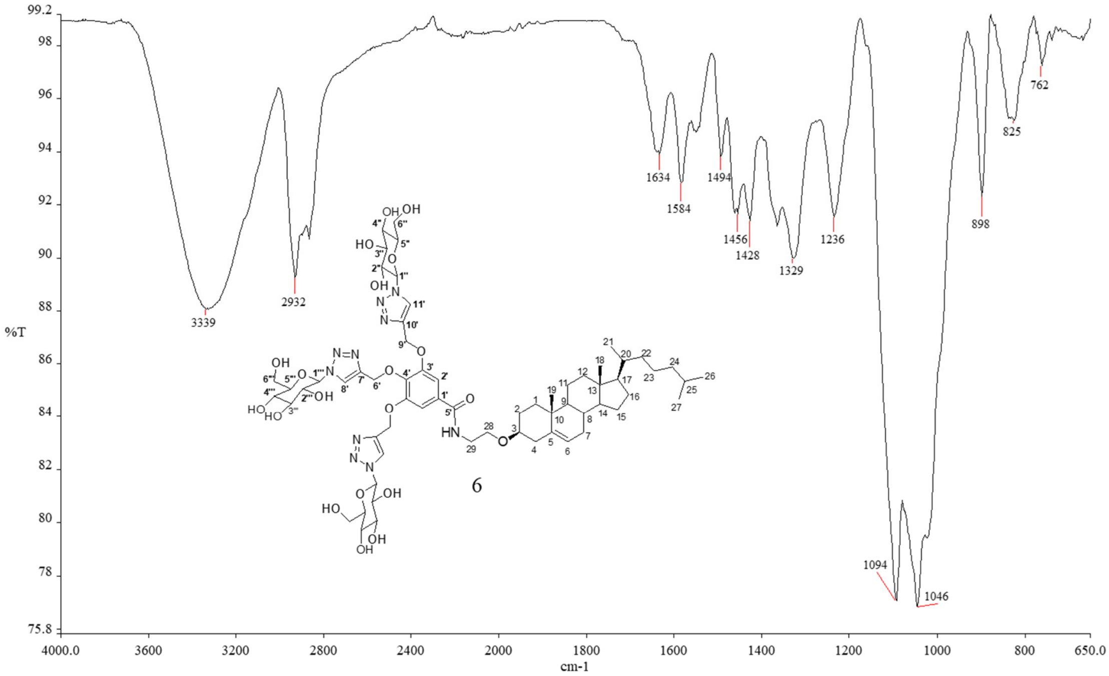

^{1}H NMR spectrum of compound 6 (400 MHz, DMSO-d_6)

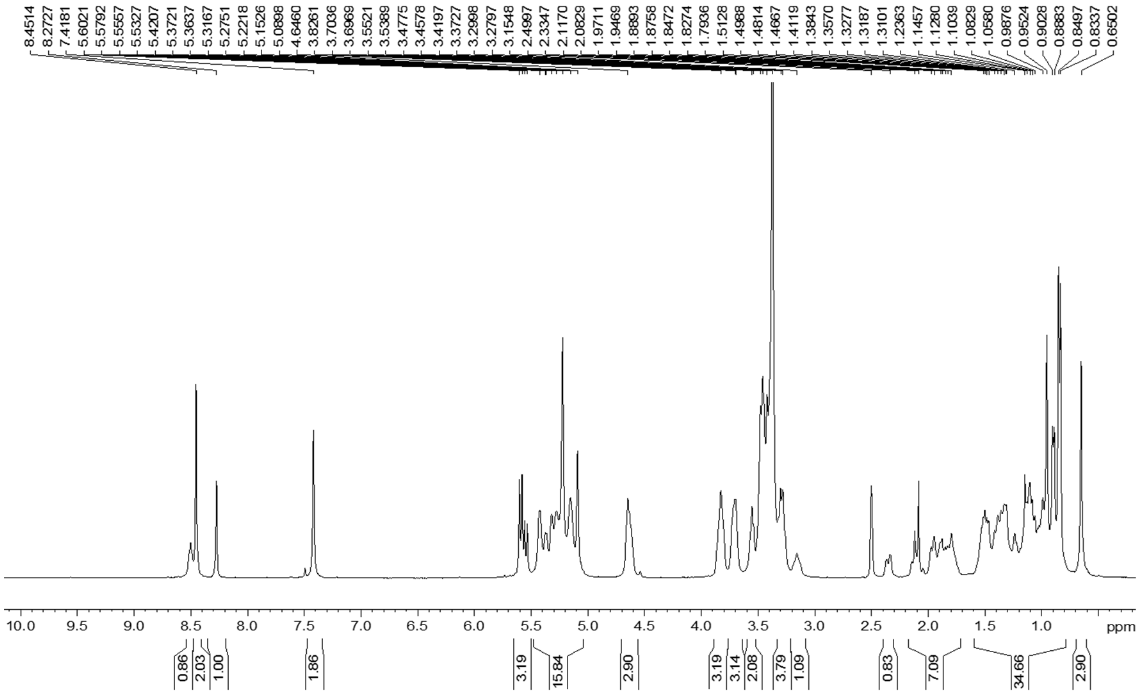

^{13}C NMR spectrum (top) and DEPT-135 (bottom) of compound 6 (100 MHz, DMSO-d_6)

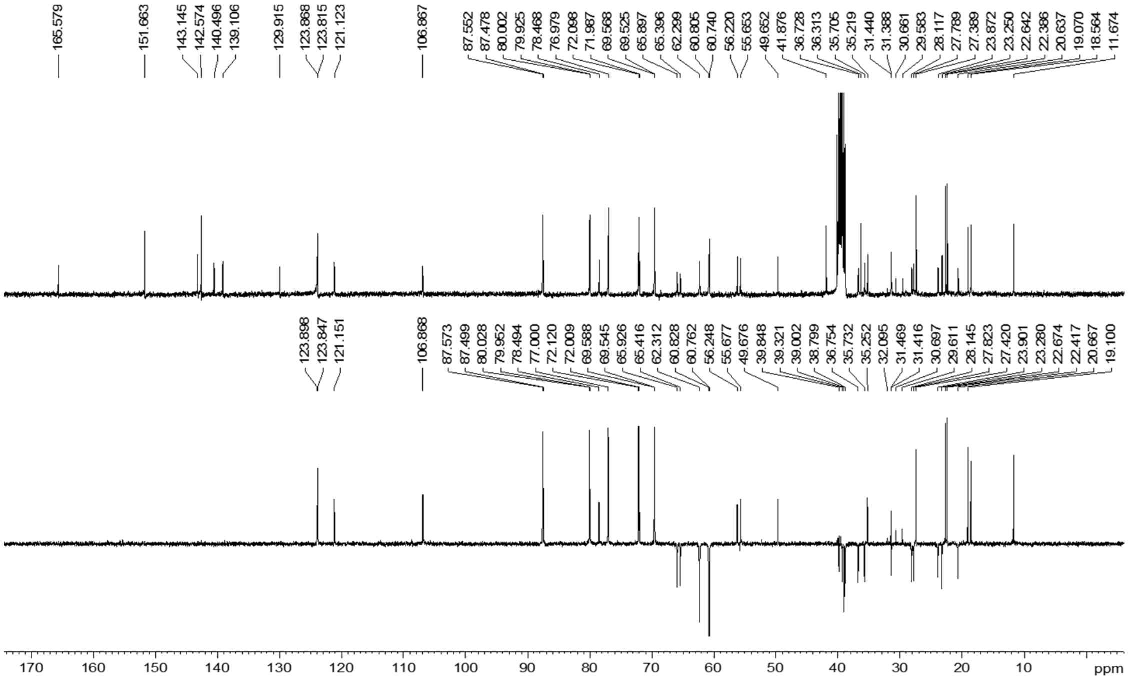

Mass spectrum (MALDI-TOF) of compound 6

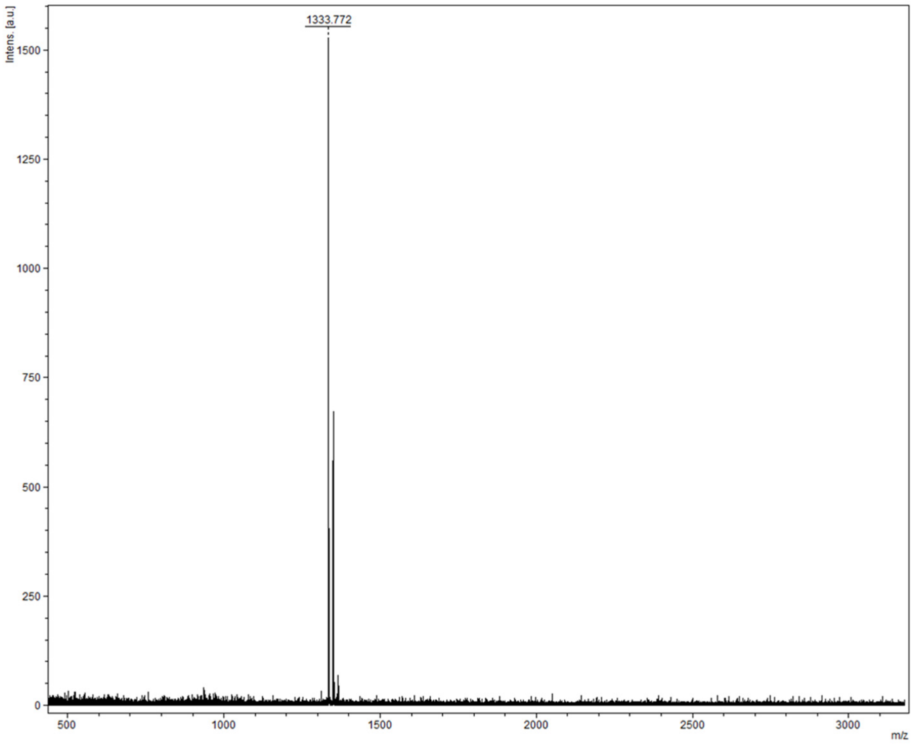

Supplement: Supplementary file 1 [file pharmaceutics-15-02751-s001.zip › pharmaceutics-2714018-supplementary.pdf]
